# Effects of physical exercise on menstrual symptoms in polycystic ovary syndrome: a systematic review

**DOI:** 10.1007/s40618-026-02836-0

**Published:** 2026-04-20

**Authors:** Marianna Molinaro, Francesca Greco, Alice Ingargiola, Antonino Raffa, Emanuela Alessandra Greco, Gian Pietro Emerenziani, Antonio Aversa

**Affiliations:** 1 Dipartimento di Medicina Sperimentale e Clinica, Università Magna Graecia, Catanzaro, Italy; 2 Dipartimento di Scienze Economiche, Psicologiche, della Comunicazione, della Formazione e Motorie, Nicolò Cusano University, Rome, 00166 Italy

**Keywords:** Women’s health, Endocrinology, Physical activity, Menstrual cycle

## Abstract

**Background:**

Despite the current guidelines, evidence on the benefits of physical exercise (PE) in PCOS remains limited and heterogeneous. While PE’s positive metabolic effects are established, its impact on menstrual symptoms is poorly understood. This review synthesises evidence on whether PE improves menstrual symptoms in reproductive-aged women with PCOS.

**Methods:**

Three databases (PubMed, Scopus, and Web of Science) were searched for peer-reviewed English articles up until March 25, 2025, without any restrictions on the publication date. The search results were conducted independently by two different reviewers, and the reference lists of the selected papers have been searched. A narrative synthesis of the included studies was conducted. The types of study selected were mainly randomised controlled trials, including High-Intensity Interval Training (HIIT) in three studies, Moderate-Intensity Continuous Training (MICT/aerobic) in seven, and combined aerobic-resistance training in two. PE lasted from 12 weeks to 6 months, primarily at 3 sessions/week. Menstrual Cyclicity/Frequency was measured in all studies via menstrual diaries.

**Results:**

Ten studies involving 290 participants with PCOS (BMI ≥ 25 kg/m²; aged 18–40 years) were included. PE improved cycle regularity across interventions. HIIT showed the highest improvement rate (69%), while MICT was effective for the most participants (*n* = 132). Protocols typically lasted ≥ 3 months at 3 sessions/week. Adding resistance training provided further benefits, particularly for women with hyperandrogenism.

**Discussion:**

The findings suggest that PE may lead to significant improvements in menstrual cyclicity. However, due to the high heterogeneity in patients’ enrolment and different intervention protocols applied with limited long-term follow-up, the reproducibility of our investigation remains to be confirmed.

**Registration:**

This systematic review was registered in PROSPERO (CRD420251009235).

## Introduction

 Polycystic ovary syndrome (PCOS) is a prevalent endocrine disorder affecting up to 15% of reproductive-aged women worldwide, characterized by clinical and/or biochemical hyperandrogenism, ovulatory dysfunction, and polycystic ovarian morphology [1]. According to Rotterdam criteria, a combination of at least two of the three criteria is required for diagnosis [2].

The pathogenesis of PCOS still remains unclear, although recent studies suggest that it is related to genetic, epigenetic, and environmental factors [3]. The clinical manifestation of PCOS includes hypersecretion of androgens, irregular menstrual cycles, hirsutism, acne, and male pattern alopecia. It is also associated with various comorbidities, including obesity, dyslipidaemia, hypertension, insulin-resistance or diabetes mellitus [4].

Among PCOS management, both pharmacological and non-pharmacological approaches can be applied [5, 6]. Pharmacological therapies aim to regulate the menstrual cycle, reduce symptoms related to hyperandrogenism and improve fertility and metabolic symptoms. Specifically, when menstrual irregularities and hyperandrogenism occur, combined oral contraceptives (COCP) are the main treatment used [7]. However, the first-line management of PCOS involves non-pharmacological therapies (i.e., lifestyle modifications) [7]. Indeed, evidence-based guidelines emphasize a multidisciplinary approach, with lifestyle intervention as a cornerstone treatment, demonstrating benefits in menstrual regulation, hyperandrogenism reduction, hyperinsulinemia management, and quality-of-life enhancement [1, 8]. Among the lifestyle modifications, guidelines highlight the need to minimize sedentary behaviour, increasing physical activity levels to optimize metabolic and reproductive health outcomes in PCOS care [9, 10]. The 2023 PCOS recommendations advocate for ≥ 150 min per week of moderate-intensity exercise or ≥ 75 min per week of vigorous-intensity exercise to prevent weight gain. For weight loss and prevention of weight regain, higher volumes of activity are suggested ≥ 250 min per week of moderate exercise or ≥ 150 min per week of vigorous exercise [7].

Despite the current guidelines, studies underlying the beneficial effects of lifestyle interventions in women with PCOS are still limited and heterogeneous [11]. Specifically, when it comes to investigate the effects of physical exercise (i.e., structured and planned physical activity) in women affected by PCOS, evidence is mainly oriented to metabolic and cardiometabolic profiles only [12, 13]. While exercise is established to improve metabolic outcomes in PCOS, its effects on menstrual symptoms remain poorly understood [14]. Moreover, the optimal type, intensity, and duration of exercise for menstrual symptom management remain debated [15]. Therefore, this systematic review aims to synthesize current evidence on exercise interventions in PCOS, with a specific focus on their effects on menstrual dysfunction. It aims to identify research gaps and clarify which exercise type may be most effective for managing PCOS-related menstrual symptoms.

## Materials and methods

### Information sources

This systematic review was registered in PROSPERO (ID number: CRD420251009235) and conducted following the Preferred Reporting Items for Systematic Reviews and Meta-Analyses (PRISMA) guidelines [16].

### Search strategy

The literature search was conducted across three different databases: PubMed, Scopus, and Web of Science. The search was conducted on March 25, 2025, on each database (Table 1). A search strategy was generated using the following terms: (PCOS-related symptoms OR polycystic ovarian syndrome OR menstrual disorders OR menstrual cycle OR menses regularity OR irregularity OR menstrual OR menstruation OR menstrual frequency) AND (exercise). The keywords were used to build a search query, specific to each database. All articles containing the searched keywords in the title, abstract, and/or keyword section were examined. A manual search of the reference list in the studies identified by the search strategy was also done.

**Table 1:** Search strategy adopted in each database

Database	Search Query
Pubmed	((((((((PCOS-related symptoms[Title/Abstract]) OR (polycystic ovarian syndrome[Title/Abstract])) OR (menstrual disorders[Title/Abstract])) OR (menstrual cycle[Title/Abstract])) OR (menses regularity[Title/Abstract] OR irregularity[Title/Abstract])) OR (menstrual[Title/Abstract])) OR (menstruation[Title/Abstract])) OR (menstrual frequency[Title/Abstract])) AND (exercise[Title/Abstract])
Scopus	(TITLE-ABS-KEY (pcos-related AND symptoms) OR TITLE-ABS-KEY ( polycystic AND ovarian AND syndrome ) OR TITLE-ABS-KEY ( menstrual AND disorders ) OR TITLE-ABS-KEY ( menstrual AND cycle ) OR TITLE-ABS-KEY ( menses AND regularity OR irregularity ) OR TITLE-ABS-KEY ( menstrual ) OR TITLE-ABS-KEY ( menstruation ) OR TITLE-ABS-KEY ( menstrual AND frequency ) AND TITLE-ABS-KEY ( exercise ) )
Web Of Science - Medline	(((((((((((((((TI=(PCOS-related symptoms )) OR AB=(PCOS-related symptoms )) OR TI=(polycystic ovarian syndrome)) OR AB=(polycystic ovarian syndrome)) OR TI=(menstrual disorders)) OR AB=(menstrual disorders)) OR TI=(menstrual cycle)) OR AB=(menstrual cycle)) OR TI=(menses regularity or irregularity)) OR AB=(menses regularity or irregularity)) OR TI=(menstrual)) OR AB=(menstrual)) OR TI=(menstruation)) OR AB=(menstruation)) OR TI=(menstrual frequency )) OR AB=(menstrual frequency ) AND (TI=(exercise)) OR AB=(exercise)

### Eligibility criteria

Articles were deemed as eligible for the review and included or excluded based on predetermined selection criteria. Inclusion criteria consisted of original articles written in English, women of reproductive age with phenotypic features of PCOS and fulfilling the Rotterdam criteria [2]; structured physical exercise intervention only associated with menstrual symptoms. Exclusion criteria consisted of adolescents < 18 years of age; menopausal women; studies that used animal models or self-reported measurements; physical exercise combined with other protocols (e.g., diet, pharmacological treatments).

### Study selection process

After the search was carried out, studies were selected from the search results based on the selection criteria, independently by two different reviewers (M.M. and A.I.). The abstracts of all articles were screened for eligibility, and a provisional selection of studies was prepared. The reviewers compared and discussed the provisional selections. In case of disagreement, a third reviewer (F.G.) was invited to provide a judgment.

### Data collection process

Data points of interest from the selected articles were collected and summarized in Table 2. Items of importance were bibliographic information (first author, year of publication), study design, participants’ characteristics, exercise intervention, menstrual outcome, and primary result.

**Table 2:** Characteristics of included studies

Author	Study design	PCOS participants (*n*)	Age (years)	Exercise Frequency - type - Intensity - Duration	Menstrual outcome	Primary result	Quality of Evidence
Benham et al. [17]	RCT	HIIT group: 16 CAET group: 14 Control group: 17	Range: 18–40	- HIIT: 10 × 30s at 90% HRR + 90s recovery, 3×/week. - CAET: 40 min moderate-intensity (50–60% HRR), 3×/week. 6 months	Menstrual regularity Tool: menstrual diary	No significant differences in cycle regularity in both exercises	Good
Kiel et al. [18]	RCT	LV-HIIT group:18 HV-HIIT group:20 Control group:20	Range: 18–45	-LV-HIIT: 10 × 1 min high-intensity -HV-HIIT: 4 × 4 min at 90–95% HRmax followed by 36 weeks of home-based HIIT (Both performed 3 times/week, semi-supervised, for 16 weeks)	Menstrual frequency Tool: Menstrual diary/Menstrual pattern questionnaires	Menstrual frequency improved after 12 months of exercise intervention	Good
Nybacka et al. [19]	RCT	Exercise group: 17	Range: 18–40 years	Individually tailored aerobic and resistance training, supervised, 2–3 times per week, 45–60 min, 4 months	Menstrual cyclicity Tool: menstrual diary	After 4 months of exercise 53% of patients had regular cycles	Good
Orio et al. [20]	Double-blind, parallel RCT	39 women	Mean: 26.1 ± 2.7	SEPT: 3×/week, 45 min/session, cycling at 60–70% VO₂ max, supervised, 6 months	Menstrual frequency (% of spontaneous vs. expected menses) Tool: menstrual diary	Improved menstrual frequency	Good
Palomba et al. [21]	Non-randomized pilot study	17 women	Mean: 26.8 ± 5.1 Range: 18–35	SEPT: 3x/week supervised cycling sessions (30 min at 60–70% VO₂max) 24 weeks	Menstrual cyclicity Tool: menstrual diary	Significant improvement of menstrual cyclicity	Fair
Patten et al. [22]	RCT	MICT (control group): 11 HIIT (intervention group): 13	Range: 18–45	– MICT: continuous training at 60–75% HR, 3×/week, 12 weeks – HIIT: >90% HR, 3×/week, 12 weeks Supervised	Menstrual cyclicity Tool: menstrual diary	HIIT reported 7.8 times higher menstrual regularity than MICT (69% vs. 22%)	Good
Redman et al. [23]	Prospective intervention trial	8 women	Range: 18–30	Supervised aerobic exercise: − 5 days/week for 16 weeks - Individualized intensity (targeting exercise energy expenditure, ExEE)	Menstrual cyclicity Tool: menstrual diary	88% resumed menses (2 + episodes)	Good
Stener-Victorin. et al. [24]	RCT	Exercise group: 5 Control group: 6	Mean: 30.4 ± 5.5 range: 25–36	Aerobic exercise (≥ 3x/week, 30–45 min, HR > 120 bpm) 16 weeks	Menstrual cyclicity Tool: menstrual diary	No change in menstrual pattern	Good
Thomson et al. [25]	RCT	Diet and aerobic exercise (DA) group: 18 Diet and combined aerobic-resistance exercise (DC) group: 20	Mean: 29.3 ± 0.7	-Aerobic: walking/jogging (5×/week, progressing to 45 min at 75–80% HRmax) -Combined: aerobic (3×/week) + resistance (2×/week, training load progressing to 65–75% 1 RM) 20 weeks	Menstrual cyclicity Tool: menstrual diary	Improved menstrual cyclicity in both exercises: 42.9% (DA), 44.4% (DC).	Good
Tiwari et al. [26]	RCT	Exercise + placebo group: 31	Mean: 24.46 ± 4.76	Marching in place, 30 min, 3 times/week for 3 months (supervised), then continued at home	Menstrual cyclicity Tool: menstrual diary	Menstrual cycle improvement was 55.17% after exercise	Good

RCT, randomised control trial; CAET, Continuous Aerobic Exercise Training; LV-HIIT, Low-Volume High-Intensity Interval Training; HV-HIIT, High-Volume High-Intensity Interval Training; HRR, Heart Rate Reserve; HR, Heart Rate; SEPT, structured exercise training program; VO2 max, Volume Oxygen Maximum; MICT, moderate-intensity continuous aerobic exercise; ExEE, Exercise Energy Expenditure; 1RM, One-Repetition Maximum; DA, Diet and aerobic exercise; DC, Diet and combined exercise

### Assessment of risk of bias in included studies

Risk of bias was evaluated by using the study quality assessment of Controlled Intervention studies [Study Quality Assessment Tools | NHLBI, NIH, Quality Assessment Tool for Observational Cohort and Cross-Sectional Studies - NHLBI, NIH. https://www.nhlbi.nih.gov/health-topics/study-quality-assessment-tools (accessed 01 August 2025)]. Two reviewers (A.I. and M.M.) independently assessed the methodological quality, and disagreements were resolved by consensus. Studies were evaluated as Good, Fair or Poor and the quality assessment results of each study are reported in supplementary file 1 (Table 3).

**Table 3:** Study quality assessment tools for each article, the response to each criterion are reported (Y = yes, N = no, NR = not reported), along with the total number of positive responses, the overall quality percentage, and the final rating (Good, fair or Poor)

Article	Q1	Q2	Q3	Q4	Q5	Q6	Q7	Q8	Q9	Q10	Q11	Q12	Q13	Q14	Tot_Yes	ApplicableQ_number	Quality percentage	Overall Score
Benham et al. [17]	Y	Y	Y	NR	NR	Y	Y	Y	NR	Y	Y	NR	Y	NR	9	9	100	Good
Kiel et al. [18]	Y	Y	Y	N	N	Y	Y	Y	N	Y	Y	Y	Y	Y	11	14	79	Good
Nybacka et al. [19]	Y	Y	NR	N	NR	Y	N	N	Y	Y	Y	Y	Y	Y	9	12	75	Good
Palomba et al. [21]	N	NA	N	N	N	Y	NR	N	Y	Y	Y	NA	Y	Y	6	11	55	Fair
Patten et al. [22]	Y	Y	NR	N	N	Y	Y	Y	Y	Y	Y	Y	Y	Y	11	13	85	Good
Redman et al. [23]	N	NA	NA	NA	NR	Y	Y	Y	Y	N	Y	NR	Y	NA	6	8	75	Good
Samadi et al. [27]	Y	NR	NR	NR	NR	Y	Y	Y	Y	Y	Y	NR	Y	Y	9	9	100	Good
Stener-victorin et al. [24]	Y	Y	Y	N	Y	Y	Y	Y	Y	Y	Y	NR	Y	NR	11	12	92	Good
Thomson et al. [25]	Y	Y	NR	N	NR	Y	N	Y	Y	Y	Y	NR	Y	Y	9	11	82	Good
Tiwari et al. [26]	Y	Y	Y	Y	Y	Y	Y	Y	Y	Y	Y	Y	Y	N	13	14	93	Good

## Results

### Study selection process

**Fig. 1:**
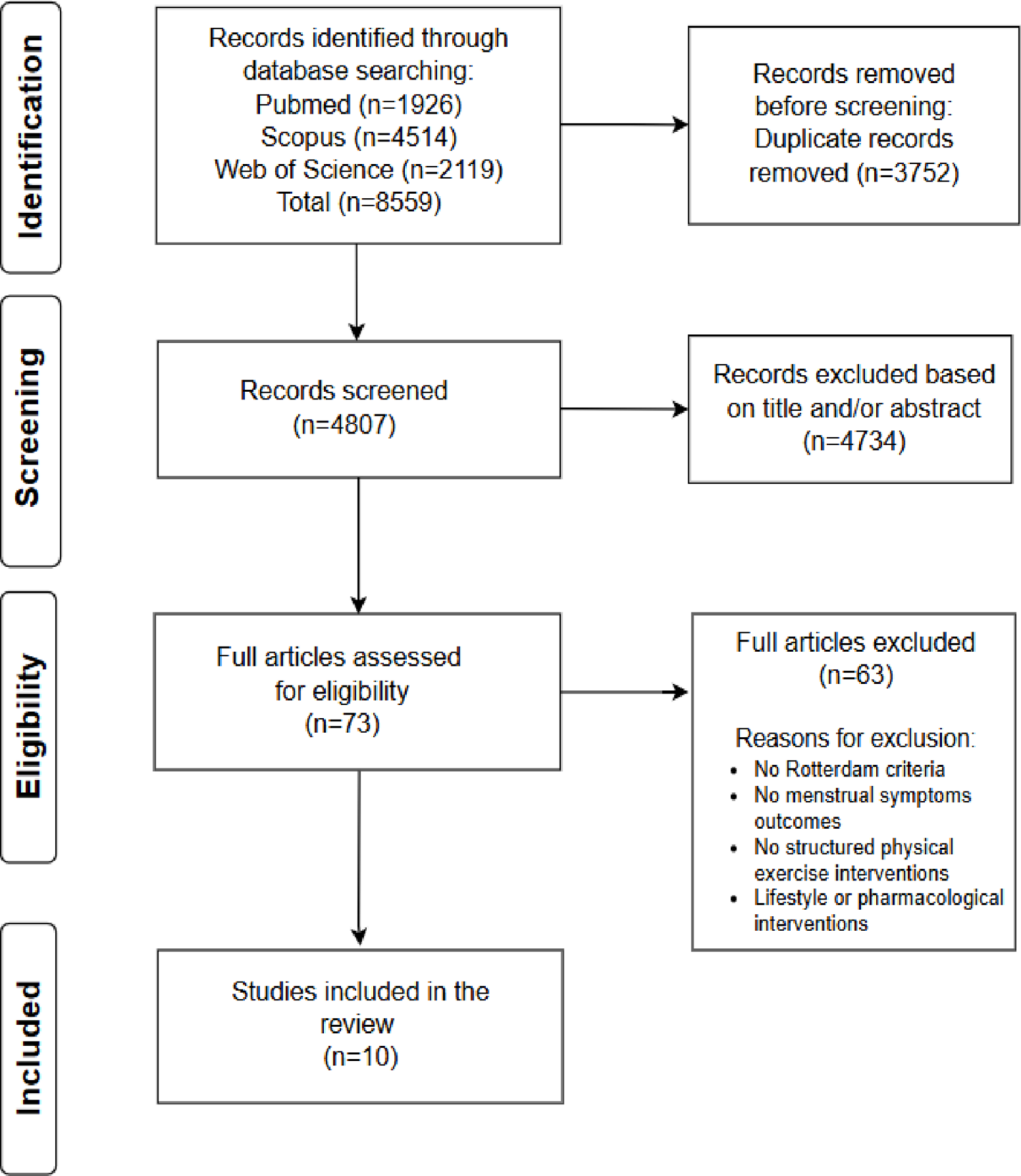
Flowchart of the study selection process

A total of 8559 articles were retrieved. Specifically, 1926 papers were found on PubMed, 4514 from Scopus, and 2119 from Web of Science by using the respective search queries for each database (Table 1). After duplicate removal, 4807 papers were initially selected, based on title and abstract, identifying 73 potential studies. After full-text screening, a total of 10 studies met the selection criteria and were included in the systematic review (Table 2). The flowchart of the study selection process is reported in Fig. 1.

### Study characteristics

#### Participants

The included articles (*n* = 10) were published between 2008 and 2022, with a total of *n* = 290 women with PCOS (BMI ≥ 25 kg/m²) [17–26]. We observed that all included studies specifically recruited women with overweight or obesity (BMI ≥ 25 kg/m²). This pattern aligns with the broader research focus on structured exercise for PCOS in this population. Consequently, this review reflects the effects of physical exercise in this specific BMI-defined population, and findings may not be generalizable to normal-weight women with PCOS due to the lack of eligible studies meeting our inclusion criteria. Most trials (*n* = 8) involved women with a mean age ≥ 30 years [17–19, 21–25].

### Exercise characteristics

Exercise interventions lasted 12–24 weeks in six articles [22, 20, 21, 23–25] and four ≥ 12 weeks in [17–19, 26].

Aerobic exercise type was used in five trials [20, 21, 23–25], HIIT (High-Intensity Interval Training) in three [17, 18, 22] and combined training (i.e., aerobic and resistance) in two [19, 25]. The frequency of exercise was mainly set at 3 sessions/week (*n* = 7) [17, 18, 20–22, 24, 26], at 5 sessions/week in two articles [23, 25] and between 2 and 3 sessions/week in 1 trial [19].

Exercise intensity was moderate in two trials [17, 22], moderate-vigorous in two [25] (combined group) [20], vigorous in one [25] (aerobic group)], near to maximal in four trials [22, 24] (HIIT group) [18], (HV-HIIT group) [17], (HIIT group)] and was not specified in three studies [19, 23, 26].

### Comparisons

Four trials compared exercise to a no-intervention control group [17, 18, 23, 24]; three trials to usual care/diet [19, 21, 25] and three with different exercise intensities and types [22] (HIIT vs. MICT) [18], (LV-HIT vs. HV-HIT) [17], (HIT vs. CAET)].

### Secondary outcomes - hormonal, metabolic and anthropometric parameters

All the information regarding the secondary outcomes are reported in Table 4. On the hormonal side, several studies saw shifts in hormone levels. For instance, hyperandrogenism improved in some cases with regular exercise programs. Patten et al. [22] found increased levels of SHBG and decreased FAI after HIIT. Thomson et al. [25] saw similar results in groups doing diet and exercise together, and Palomba et al. [21] noticed it in women who were ovulating after structured training. However, there are studies like the ones of Stener-Victorin et al. [24] and Redman et al. [23] where aerobic exercise did not change testosterone or SHBG. AMH dropped in the low-volume HIIT group after 16 weeks, according to Kiel et al. [18].

Metabolic parameters showed more consistent positives from exercise. Insulin sensitivity improved in studies with aerobic exercise [20, 21, 25], HIIT [22], or mixed training [25]. Orio et al. [20] revealed an improvement in lipid profiles like lower total and LDL cholesterol, higher HDL, and less inflammation with CRP and PAI-1 after cycling sessions. In contrast, Benham et al. [17] did not find changes in HOMA2-IR, and in Kiel et al. [18] study HOMA-IR increased in the high-volume HIIT at 12 months.

Anthropometric changes were not always the same. BMI and waist size dropped in studies mixing exercise with diet [25] or just aerobic training [20, 21, 24, 26]. Interestingly, Patten et al. [22] and Redman et al. [23] showed metabolic benefits regardless of relevant weight loss. Kiel et al. [18] mentioned more fat-free mass in high-volume HIIT at 12 months, but fat mass stayed the same.

### Risk of bias

Nine studies were reported as having good quality - low risk [17–19, 22–27], whereas only one as a fair quality - medium risk [21].

**Table 4:** Detailed characteristics of included studies on secondary outcomes (hormonal, metabolic, anthropometric)

Author	Study design	PCOS participants (*n*)	Age (years)	Exercise Frequency - type - Intensity - Duration	Hormonal and biochemical outcomes	Anthropometric parameters	Exercise effect
Benham et al. [17]	RCT	HIIT group: 16 CAET group: 14 Control group: 17	range: 18–40	- HIIT: 10 × 30s at 90% HRR + 90s recovery, 3×/week. - CAET: 40 min moderate-intensity (50–60% HRR), 3×/week. 6 months	- **Fasting Glucose**: Significantly increased in HIIT vs. Control (+ 0.3 mmol/L, *p* = 0.02). **Fasting Insulin**: Significantly increased in Control group (+ 19.5 mIU/L, *p* = 0.04). No significant changes in HIIT or CAET. **HOMA2-IR**: No significant differences. **HbA1c**: No significant differences. **Androgens**,** SHBG**: not evaluated	**BMI**:significant decrease in CAET vs. Control (-1.0 kg/m², *p* < 0.01) and vs. HIIT (-0.9 kg/m², *p* = 0.04). **Waist circumference**: Significant decrease within ALL groups (HIIT: -7.3 cm; CAET: -6.9 cm; Control: -4.5 cm).No significant differences between groups.	No significant differences in cycle regularity in both exercises
Kiel et al. [18]	RCT	LV-HIIT group:18 HV-HIIT group:20 Control group:20	range: 18–45	-LV-HIIT: 10 × 1 min high-intensity -HV-HIIT: 4 × 4 min at 90–95% HRmax followed by 36 weeks of home-based HIIT (Both performed 3 times/week, semi-supervised, for 16 weeks)	**SHBG**: reduced in HV-HIT vs. control (*p* = 0.035) and vs. LV-HIT (*p* = 0.024) at 12 months **AMH**: reduced in LV-HIT at 16 weeks vs. baseline (*p* = 0.014) and vs. control (*p* = 0.015) and HV-HIT (*p* = 0.024) **FG score**: reduced in HV-HIT vs. control (*p* = 0.005) and vs. LV-HIT (*p* = 0.047) at 12 months **Testosterone**,** androstenedione**,** 17OH-P**: No between-group differences **HOMA-IR**: increased in HV-HIT vs. LV-HIT at 12 months (*p* = 0.032) **Glucose**,** insulin**,** lipids**: No significant between-group differences at 16 wk or 12 mo	**Weight**,** BMI**,** waist circumference**,** fat mass**,** fat percentage**,** visceral fat**: No significant between-group differences at 16 wk or 12 mo **Fat-free mass**: increased in HV-HIT at 12 mo vs. baseline (*p* < 0.05) **Waist/hip ratio**: No significant changes	Menstrual frequency improved after 12 months of exercise intervention
Nybacka et al. [19]	RCT	Exercise group: 17	range: 18–40 years	Individually tailored aerobic and resistance training, supervised, 2–3 times per week, 45–60 min, 4 months	**AMH**: no change in Exercise group. **LH**,** FSH**,** SHBG**: No significant changes	**BMI**: reduced in exercise groups (*p* < 0.05) **Upper/lower body fat**,** lean body mass**: Variable changes	After 4 months of exercise 53% of patients had regular cycles
Orio et al. [20]	Double-blind, parallel RCT	39 women	mean: 26.1 ± 2.7	SEPT: 3×/week, 45 min/session, cycling at 60–70% VO₂ max, supervised, 6 months	**Free T**,** FAI**,** FG score**: no significant changes **SHBG**,** DHEAS**,** 17-OHP**: No significant changes **HOMA-IR**,** fasting insulin**,** and AUCins**: were reduced in the SETP group (*p* < 0.05) **GIR** improved in SETP (*p* < 0.05). **Fasting glucose**,** AUCglu**: No changes	**BMI**: reduced in SETP group (*p* < 0.05), **WHR**: reduced in SETP group (*p* < 0.05)	Improved menstrual frequency
Palomba et al. [21]	Non-randomized pilot study	17 women	Mean: 26.8 ± 5.1 Range: 18–35	SEPT: 3x/week supervised cycling sessions (30 min at 60–70% VO₂max), 24 weeks	**Testosterone**: reduced in ovulatory patients (*p* < 0.05) **SHBG**: increased in SET ovulatory patients (*p* < 0.05) **FAI**: increased in ovulatory patients in SET group (*p* < 0.05) **Androstenedione**,** DHEA-S**: no change in SET group **Fasting insulin**: reduced in ovulatory patients in SET groups (*p* < 0.05) **GIR**: inreased in SET ovulatory patients (*p* < 0.05) **HOMA-IR**: reduced in ovulatory patients (*p* < 0.05) **Other hormones (LH**,** FSH**,** 17-OHP**,** etc.)**: No significant changes	**Weight**,** BMI**,** WC**: reduced in ovulatory patients (*p* < 0.05) **WHR**: reduced in SET groups (*p* < 0.05) **FG score**: No significant changes	Significant improvement of menstrual cyclicity
Patten et al. [22]	RCT	MICT (control group): 11 HIIT (intervention group): 13	range: 18–45	– MICT: continuous training at 60–75% HR, 3×/week, 12 weeks – HIIT: >90% HR, 3×/week, 12 weeks Supervised	**SHBG**: HIIT significantly increased SHBG versus MICT. **FAI**: HIIT intervention markedly reduced FAI levels. **AMH**,** androstenedione**,** estradiol and DHT**: were not altered by either intervention **Insulin sensitivity index (ISI)**: HIIT increased ISI significantly compared to MICT. **Fasting glucose**: HIIT significantly reduced resting fasting glucose.	**Weight/ BMI** : no observed changes in weight or BMI as a result of either interventio **Waist circumference**: Both the HIIT and MICT groups decreased waist circumference (β = -0.98 cm, *P* < 0.02n.	HIIT reported 7.8 times higher menstrual regularity than MICT (69% vs. 22%)
Redman et al. [23]	Prospective intervention trial	8 women	range: 18–30	Supervised aerobic exercise: − 5 days/week for 16 weeks - Individualized intensity (targeting exercise energy expenditure, ExEE)	**Testosterone**,** SHBG**,** Free Androgen Index (FAI)**: Exercise training did not change total testosterone or sex hormone binding globulin **Insulin sensitivity**: was significantly increased from baseline by 34 ± 6% (*p* < 0.001)	**Weight**: no observed changes in weight after 16 week of exercise **Waist circumference and BMI**: not valuated	88% resumed menses (2 + episodes)
Stener-Victorin. et al. [24]	RCT	Exercise group: 5 Control group: 6	Mean: 30.4 ± 5.5 range: 25–36	Aerobic exercise (≥ 3x/week, 30–45 min, HR > 120 bpm) 16 weeks	**Testosterone**,** SHBG**,** Free Androgen Index (FAI)**,** Insulin sensitivity**: No significant difference.	**Weight/ BMI**: exercise significantly reduced both.	No change in menstrual pattern
Thomson et al. [25]	RCT	Diet and aerobic exercise (DA) group: 18 Diet and combined aerobic-resistance exercise (DC) group: 20	mean: 29.3 ± 0.7	-Aerobic: walking/jogging (5×/week, progressing to 45 min at 75–80% HRmax) -Combined: aerobic (3×/week) + resistance (2×/week, training load progressing to 65–75% 1 RM) 20 weeks	**Testosterone**: Significant decrease in all groups. **SHBG**: Significant increase in all groups. **Free Androgen Index (FAI)**: Significant decrease in all groups. **Fasting Insulin**: Significant decrease in all groups. **Insulin Resistance (HOMA)**: Significant decrease in all groups. **Fasting Glucose**: Significant decrease in all groups. There was no significant difference in outcomes between the treatment groups	**Weight**: Decreased in all groups (~ 9.4%). No difference between treatments. **Waist Circumference)**: Decreased in all groups (~ 11.1%). No difference between treatments.	Improved menstrual cyclicity in both exercises: 42.9% (DA), 44.4% (DC).
Tiwari et al. [26]	RCT	exercise + placebo group: 31	Mean: 24.46 ± 4.76	Marching in place, 30 min, 3 times/week for 3 months (supervised), then continued at home	**Testosterone**: No significant reduction at 6 months.	**Weight**: The loss was 0.78 ± 0.19 kg after 3 months and 1.08 ± 0.30 kg after 6 months **BMI**:Significant reduction at 3 and 6 months.(*p* < 0.0001) **Waist circumference**: Significant decrease, ~ 3 cm total.(*p* < 0.0001)	Menstrual cycle improvement was 55.17% after exercise

**PCOS**: polycystic ovary Syndrome; **RCT**: randomized controlled Trial; **HIIT**: High-Intensity interval training; **LV-HIIT**: Low-Volume HIIT; **HV-HIIT**: High-Volume HIIT; **CAET/MICT**: continuous aerobic exercise training / Moderate-Intensity continuous training; **HRR**: heart rate Reserve; **HR/HRmax**: heart rate / Maximum heart rate; **VO₂ max**: maximal oxygen Uptake; **SEPT**: structured exercise training Program; **1RM**: One-Repetition Maximum; **DA**: diet and aerobic exercise; **DC**: diet and combined exercise (aerobic and resistance); **SHBG**: sex Hormone-Binding Globulin; **FAI**: free androgen Index; **AMH**: Anti-Müllerian Hormone; **FG score**: Ferriman-Gallwey score; **DHEA-S**: dehydroepiandrosterone Sulfate; **17-OHP**: 17-hydroxyprogesterone; **LH**: luteinizing Hormone; **FSH**: Follicle-Stimulating Hormone; **HOMA-IR**: homeostatic model assessment of insulin Resistance; **ISI**: insulin sensitivity Index; **AUCins/AUCglu**: area under the curve for insulin/Glucose; **GIR**: glucose infusion rate; **HbA1c**: glycated Hemoglobin; **TC**: total Cholesterol; **LDL-C**: LDL Cholesterol; **HDL-C**: HDL Cholesterol; **CRP**: C-Reactive Protein; **PAI-1**: plasminogen activator Inhibitor-1; **BW**: body Weight; **BMI**: body mass Index; **WC**: waist Circumference; **WHR**: waist-to-Hip ratio

## Discussion

In recent years, growing attention has been directed toward the potential of exercise as a therapeutic strategy to improve menstrual regularity in women with polycystic ovary syndrome (PCOS). At present, the evidence is quite mixed, with results differing based on the type, intensity, and duration of the exercise, as well as on the individual characteristics of the participants [28]. This review suggests that engaging in physical exercise can be a valuable method for enhancing menstrual regularity in women with PCOS, regardless of any weight loss. In most studies included, exercise was linked to either an increase in ovulation frequency or a decrease in cycle length.

### Aerobic exercise

Concerning aerobic exercise, moderate-intensity continuous aerobic exercise (MICT or CAET) is a well-established intervention for improving menstrual function in women with PCOS [29]. Orio et al. [20] showed that structured exercise training programs (SEPT) significantly boosted the frequency of menstrual cycles. Thomson et al. [25] demonstrated that only 42.9% (9 of 21) of women affected by PCOS reported improvements in menstrual cyclicity after undergoing a walking/jogging aerobic program performing five sessions per week. In contrast, Ujvari et al. [30] reported that 65% of participants showed improved menstrual cyclicity after a 3-month lifestyle intervention that included an aerobic exercise program alongside a dietary component; this combined intervention also led to an increase in endometrial insulin signalling markers. These results are strongly supported by Redman et al. [23], where 88% of women with PCOS returned to regular cycles after 16 weeks of supervised aerobic exercise, accompanied by a reduction in ovarian follicle count on MRI. Palomba et al. [21] found that structured exercise training alone led to higher menses frequency (*p* = 0.043) and ovulation rates, indicating that exercise might provide unique advantages beyond diet al.one. These results are strongly supported by the study of Vigorito et al. [31] where vigorous aerobic activities (e.g., cycling or running at 60–70% VO₂ max/three times a week) improved menstrual regularity, regardless weight loss, primarily by improving insulin sensitivity [32]. Therefore, aerobic exercise may represent an important non-drug approach to significantly easing menstrual symptoms and helping restore ovarian function in women dealing with PCOS.

### Combined exercise (aerobic and resistance training)

The combination of aerobic and resistance exercise may potentiate improvements in reproductive function by addressing different physiological pathways. Eight weeks of combined training have demonstrated improvements in menstrual cyclicity without substantial weight loss [33]. However, Thomson et al. [25] reported that the addition of aerobic or combined exercise to a hypocaloric diet did not lead to further improvements in menstrual cyclicity in women with severe obesity, where losing about 10% of body weight was the main factor in restoring ovulation. This indicates that for women with severe obesity, weight loss remains the primary intervention for restoring menstrual function, while physical exercise is mandatory since it can help maintaining lean mass and improve body composition. Finally, Nybacka et al. [19] demonstrated that, after four months of individually tailored aerobic and resistance training 2–3 times per week, 53% of patients had regular cycles.

### High-intensity interval training (HIIT)

HIIT has emerged as a potent exercise modality, particularly for its metabolic benefits. The study by Patten et al. [22] offers some compelling evidence that HIIT can help to restore menstrual cycles. In this randomized controlled trial, women who participated in the HIIT sessions experienced a notable improvement in menstrual regularity: 69% compared to just 22% in the moderate-intensity continuous training (MICT) group. This improvement was linked to better insulin sensitivity, increased levels of sex hormone-binding globulin (SHBG), and a drop in the free androgen index (FAI). These findings align with Kiel et al. [18], whose IMPROV-IT trial revealed that while both low-volume and high-volume HIIT didn’t significantly improve menstrual frequency (the primary outcome), the low-volume HIIT group showed significantly higher pregnancy rates (33% vs. 0% in controls). This suggests that HIIT may enhance fertility outcomes even without dramatic changes in cycle regularity. In contrast, Benham et al. [17] found no significant differences between HIIT and Continuous Aerobic Exercise Training (CAET) regarding menstrual cycle regulation, a result potentially attributed to lower adherence to the exercise regimen (68%). Similarly, Samadi et al. [27], studied Aquatic HIIT (AHIIT) and observed a more significant improvement in menstrual cycle length compared to the control group, suggesting that AHIIT may be an effective and well-tolerated approach. However, Samadi et al. [27] combined the exercise intervention with metformin administration. High-intensity interval training (HIIT) seems to be especially beneficial for obese women with PCOS, while moderate combined aerobic-resistance training appears to be enough to quickly improve menstrual regularity in non-overweight women, likely working more through metabolic pathways than hormonal mechanisms [20, 23, 34].

### Other protocols

Alternative and complementary exercise methods offer viable options for patients unable to engage in traditional protocols. For instance, Gorczyca et al. [35] conducted a remotely delivered lifestyle intervention, combined diet, exercise (≥ 225 min/week of moderate-vigorous activity) and behavioural support with weekly video sessions focusing on goal-setting and self-monitoring, and found that 67% of participants resumed ovulation and significant improvement in menstrual problems (PCOSQ domain: *p* = 0.04), including reduced irregularity and pain, with high participant satisfaction despite moderate adherence, which really underscores the importance of flexible and accessible telehealth strategies. In another study by Stener-Victorin et al. [24], they found that in the physical exercise group, four of five were experiencing oligo-amenorrhea, and the intervention did not affect the menstrual bleeding pattern. Interestingly, only electroacupuncture seemed to improve menstrual regularity in these women, possibly by reducing sympathetic nervous system activity. Although exercise alone has proven to be effective in improving self-reported menstrual symptoms, some studies compared it with other types of intervention supplements. Among these, Tiwari et al. [26] demonstrated that aerobic exercise combined with metformin led to menstrual cycle normalization in 83.3% of cases, compared to 55.2% in the exercise-only group. This supports the hypothesis that correcting insulin resistance through both exercise and pharmacotherapy can help to restore menstrual regularity [36]. Zhang et al. [37] found that when metformin and clomiphene were combined with exercise interventions, the rates of menstrual recovery soared to 94.12%, compared to 64% in groups that relied solely on medication. An alternative approach was evaluated by Arentz et al. [15], who combined exercise with herbal supplements (e.g., Cinnamomum verum, Glycyrrhiza glabra). Their study reported a significant reduction in oligomenorrhea days and a 55% improvement in menstrual regularity, likely due to the anti-androgenic and insulin-sensitizing properties of the herbs used [15].

The experience of the menstrual cycle, with its procession of bleeding, pain, exhaustion, and emotional swings, can profoundly burden a woman’s life, impacting not only her physical health but also her psychological balance and the serenity in engaging with relationships [38]. The manifestation of menstrual symptoms can be influenced by a plenty of factors, including an inadequate diet, a lack of physical activity, the consumption of alcoholic beverages and tobacco use, as well as states of anxiety and psychological tension [39]. Evidence shows that engaging in aerobic exercise can effectively alleviate common physical menstrual symptoms and painful periods, such as bloating, nausea, hot flashes, and food cravings [40]. Joint Clinical Recommendations includes aerobic training, including vigorous activities (e.g., cycling or running at 60–70% VO₂ max /three times a week) [31] that has consistently been shown to improve menstrual regularity, even without significant weight loss [41, 42], , associated with dietary strategies [7] and motivational counselling [43] constitutes a cornerstone intervention in the management of PCOS, thereby enhancing the quality of life for women with PCOS who experience menstrual-related comorbidities, including not only oligo-amenorrhea but also physical symptoms such as pain and fatigue, which can significantly impair daily functioning and overall well-being. Overall, alternative or complementary approaches offer viable options for patients who are unable or unwilling to engage in high-intensity or aerobic exercise, and may still provide meaningful benefits in menstrual regulation, pain reduction, and overall quality of life. The review by Kazemi et al. [44] shows that women with PCOS have suboptimal diets (low in magnesium and high in cholesterol) and reduced physical activity. The PCOS phenotype may influence how women respond to exercise; for instance, those with significant insulin resistance should focus on vigorous aerobic workouts combined with dietary changes, while women with hyperandrogenism might benefit more from progressive resistance training and magnesium supplementation if they are deficient [45]. Personalization of exercise programs is essential, taking into account individual PCOS phenotypes, including obesity, hyperandrogenism, oligo-amenorrhea or insulin resistance, as well as individual preferences and tolerability.

#### Effects on secondary outcomes

The observed improvements in markers of hyperandrogenism, such as increased SHBG and decreased FAI, particularly noted with HIIT [22] and structured aerobic training [21, 25], provide a plausible endocrine mechanism for the restoration of menstrual regularity. Androgen excess disrupts follicular development and ovulation. By improving insulin sensitivity, exercise may reduce ovarian androgen production and increase hepatic synthesis of SHBG, thereby lowering bioavailable testosterone and mitigating its inhibitory effects on the menstrual cycleInsulin resistance is a central driver of both metabolic and reproductive dysfunction in PCOS. The improvements in HOMA-IR, fasting insulin, and insulin sensitivity indices [20–22, 25] strongly support the hypothesis that exercise ameliorates menstrual dysfunction primarily through this metabolic pathway. This is further reinforced by studies where menstrual improvement occurred independent of weight loss [22, 23], pointing to direct insulin-sensitizing effects of physical activity.

The impact on body composition, while variable, adds another layer to the therapeutic profile of exercise. For women with overweight or obesity, reductions in BMI and waist circumference, often achieved with combined diet and exercise interventions [25], contribute to overall metabolic health. Crucially, exercise helps to preserve or increase fat-free mass [18], which is beneficial for long-term metabolic rate and functional capacity. The finding that significant menstrual and metabolic benefits can occur even without weight loss [22, 23] is clinically empowering, as it emphasizes the intrinsic value of exercise behavior itself.

The changes in other metabolic parameters, such as lipid profile and inflammatory markers (e.g., reduced CRP) [20], suggest that exercise confers broader cardiometabolic benefits, which is highly relevant given the increased long-term health risks associated with PCOS.

Overall, analyzing these secondary outcomes reinforces and clarifies the role of exercise as a foundational therapy in PCOS. This evidence supports a personalized management approach where exercise prescriptions are tailored not only to improve menstrual cyclicity but also to address an individual’s specific dominant PCOS phenotype, whether it is hyperandrogenism, insulin resistance, or obesity, thereby optimizing comprehensive health outcomes.

### Limitations and future directions

Most effective exercise interventions highlighted in the literature are characterized by a minimum duration of 12 weeks, ranging from three to five sessions per week. However, the methodological quality of studies remains heterogeneous. While several well-structured randomized controlled trials (RCTs) [25, 26] strongly support the benefits of exercise in PCOS, other studies are limited by small sample sizes, insufficient statistical power, and wide variability in outcome measures, including ovarian morphology, documented ovulation, or menstrual diary data, making direct comparisons across studies challenging [23]. Future research should aim to uncover the molecular mechanisms through which exercise exerts its effects in PCOS, to develop targeted, phenotype-specific training protocols. Beyond improving insulin sensitivity and modulating androgen levels, other proposed pathways deserve exploration. For instance, regular physical activity is known to reduce chronic low-grade systemic inflammation, a condition often present in PCOS which can contribute to ovarian dysfunction and anovulation [46]. The low-grade chronic inflammation in PCOS patients is mainly attributed to accumulated visceral fat, in which adipocytes undergo necrosis after hypoxia and gather a large number of inflammatory cells to produce a variety of inflammatory cytokines [47]. Exercise-induced reductions in pro-inflammatory cytokines (e.g., TNF-α, IL-6) and adipokines, alongside increases in anti-inflammatory myokines, may create a more favourable intraovarian environment, potentially facilitating follicular development and ovulation. In support of this, a study in PCOS rats found that exercise for eight weeks improved polycystic ovarian morphology and decreased the levels of inflammation, OS, and fibrosis by suppressing the pro-inflammatory IRE1α-TXNIP/ROS-NLRP3 pathway [48]. Simultaneously, enhancing inflammatory medium secretion can aggravate chronic inflammation, affect the tissue cell function, and result in ovarian dysfunction and metabolic disorder [47]. Moreover, obesity-mediated neuroinflammation in the hypothalamus, characterized by microglia activation, macrophage infiltration, and increased cytokines like LIF, represses GnRH gene expression, leading to reduced LH and central dysregulation of the reproductive axis [49]. Chronic inflammation caused by obesity affects GnRH neurons, resulting in reduced levels of LH and impaired menstrual cyclicity [49]. Therefore, the attenuation of this inflammatory state, whether through exercise or weight loss, is critical for restoring hypothalamic-pituitary function. Reduction of the inflammatory burden likely contributes to the restoration of ovarian morphology and the normalization of estrous cycles observed in animal models following intervention. Furthermore, exercise influences the autonomic nervous system, potentially reducing sympathetic overactivity which is implicated in PCOS pathogenesis [50]. Elucidating these and other mechanisms (e.g., effects on oxidative stress, gut microbiota) will provide a more comprehensive understanding of how exercise benefits menstrual health in PCOS.

Large-scale, adequately powered trials employing standardized intervention protocols, rigorous outcome measures (e.g., validated menstrual questionnaires), and long-term follow-up to assess the lasting effects on menstrual cyclicity, fertility, metabolic health, and quality of life. Collectively, these findings highlight the importance of adopting personalized, multimodal strategies in PCOS management, with exercise acting as a versatile and adaptable foundational intervention that can be customized to meet each patient’s unique needs and treatment objectives.

#### Implications for clinical practice

The findings of this systematic review highlight the central role of structured physical exercise in managing menstrual symptoms associated with PCOS. For effective translation into clinical practice, it is essential that clinicians adopt a personalized approach that goes beyond generic physical activity recommendations. Prescriptions should be clear and evidence-based, precisely specifying the Type, Intensity, Duration, and Frequency (TIDF) of exercise. Moderate-intensity continuous aerobic training (MICT) forms the foundation of therapy, with sessions at 50–70% of maximum heart rate. High-intensity interval training (HIIT) represents a valid and efficient alternative for motivated patients, particularly effective in improving insulin sensitivity. The integration of strength training, especially for women with hyperandrogenism, and the adoption of combined aerobic-resistance protocols amplify overall benefits. A minimum commitment of three weekly sessions of 30–60 min each, sustained for at least 12 weeks, is necessary to achieve clinically significant improvements.

The sustainability of the intervention depends on addressing adherence barriers. Clinicians must recognize and manage challenges such as fatigue, time constraints, economic limitations, and psychosocial issues related to body image. Strategies such as starting with achievable goals, proposing efficient alternatives like HIIT, promoting low-cost activities, and fostering supportive environments are essential to building and maintaining exercise habits. In this context, the clinical approach should be structured as a true therapeutic prescription, articulated in phases of initial assessment, collaborative planning, gradual progression, and continuous monitoring through regular follow-ups.

Clinical practice recommendations must evolve toward a targeted and sustainable prescription model to maximize the impact of physical exercise in PCOS management.

## Conclusions

**Fig. 2:**
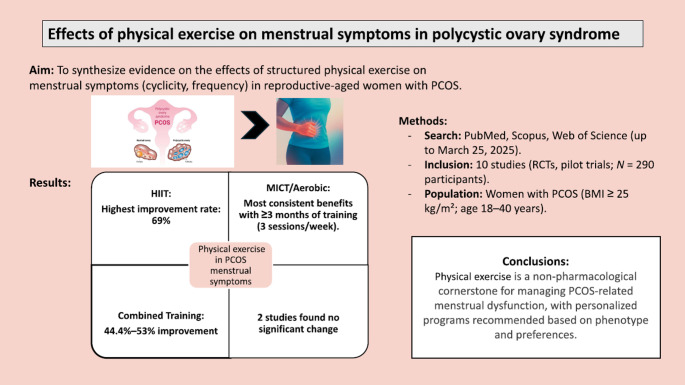
Graphical summary of the effects of physical exercise on menstrual symptoms in women with PCOS

Results show that physical exercise alone may represent a crucial therapeutic tool for managing PCOS menstrual symptoms. As illustrated in Fig. 2, it has positive impacts on menstrual regularity, related symptoms, and overall quality of life, regardless of significant weight loss. Among various exercise types, aerobic training at a moderate to vigorous intensity, typically lasting ≥ 3 months at a frequency of three times a week, has been the most thoroughly researched and effective for improving insulin sensitivity, which is a major factor in PCOS, and for restoring menstrual cycles independently from weight loss. However, the best approach is a personalized, multimodal one: adding resistance training can boost benefits, particularly for those dealing with hyperandrogenism, while flexible options like HIIT or online programs provide accessible alternatives.
